# The feasibility of culturally adapted computerised cognitive remediation for first-episode psychosis

**DOI:** 10.1192/bjo.2024.854

**Published:** 2025-03-18

**Authors:** Claire Press, Jordan Bamford, Laoise Renwick, Melissa Noke, Richard Drake, Nusrat Husain

**Affiliations:** Division of Psychology & Mental Health, Faculty of Biology, Medicine & Health, University of Manchester, Manchester, UK

**Keywords:** Psychotic disorders/schizophrenia, psychosocial interventions, transcultural psychiatry, psychological treatments, community mental health teams

## Abstract

**Background:**

British South Asians have a greater incidence of psychotic illness, which is associated with cognitive deficits. Computerised cognitive remediation aims to improve cognition.

**Aims:**

We aimed to culturally adapt computerised cognitive remediation for British South Asians with first-episode psychosis, and assess its feasibility.

**Method:**

Qualitative interviews were analysed using thematic analysis to guide cultural adaptation of cognitive remediation, followed by a case series to determine feasibility. Our sample comprised 20 participants: ten in the qualitative interviews and ten in the feasibility evaluation. The sample was generated via purposive sampling from early intervention services in England, and was an entirely Muslim cohort, who were mainly Pakistani and born in the UK. Our intervention was computerised interactive remediation of cognition training for schizophrenia (CIRCuiTS), which was culturally adapted based on formative qualitative interviews and using an established framework. Participants engaged with 40 h of tasks over 12 weeks, with the aim of improving attention, memory and executive functioning. Feasibility was explored by assessing acceptability, engagement and retention in the study, and a range of measures were used to assess impact on cognition and mental state.

**Results:**

The cultural adaptation of CIRCuiTS was found to be acceptable, with high levels of engagement and satisfaction. Despite the small sample, the intervention led to improved cognition and mental state.

**Conclusions:**

This is the first study to culturally adapt computerised cognitive remediation for British South Asians who are Muslim, and it had high acceptability with good retention, engagement and satisfaction. Future effectiveness testing is recommended.

First-episode psychosis (FEP) refers to individuals who are early in the course of a psychotic illness or treatment.^1^ Such psychotic illnesses include schizophrenia, which is a leading cause of global disability-adjusted life-years.^2^ Psychosis refers to the presence of ‘positive symptoms’ such as hallucinations and delusions, and ‘negative symptoms’ such as affective flattening, alogia, apathy, asociality and associated cognitive dysfunction.^3^ Negative symptom burden predicts prospective long-term functioning,^4^ and there is often little improvement in negative symptoms over time.^5^ Cognitive impairment is present in over 80% of those with a diagnosis of schizophrenia.^6^ Unfortunately, mainstay current pharmacological and psychological treatment for psychosis show little improvement in negative symptoms and cognitive function.^7^

## Psychosis and ethnicity

Ethnicity and migration independently predict incidence of psychotic disorders, with recent meta-analytic evidence revealing a relative risk for non-affective psychotic disorders among migrants of 2.13 (95% CI 1.99–2.27), and for affective psychotic disorders it is even higher (2.94, 95% CI 2.28–3.79), even when socioeconomic confounders have been taken into account.^8^ British South Asians have an apparent increased incidence of psychosis compared with the White British population in the UK; research examining ethnic inequalities in the incidence of schizophrenia in England found that South Asian groups have a relative risk of 2.27 (95% CI 1.63–3.16) compared with White British populations.^9^ Concerningly, in the UK, some tentative evidence suggests an overrepresentation of ethnic minorities with more severe mental illness.^10^

Cultural background can influence idioms of distress, evaluation of symptoms, engagement with services and outcomes from interventions.^11,12^ Psychosocial interventions form part of the management plan for patients with FEP.^13^ Considering psychosocial interventions for ethnic minorities, there is evidence of greater drop-out for ethnic minority groups compared with ethnic majority groups.^14^ However, qualitative evidence has described how cognitive–behavioural therapy, for example, can be an acceptable treatment if culturally adapted by including culturally based patient health beliefs, attributions concerning psychosis, attention to help seeking pathways and technical adjustments.^15^ This has been demonstrated in a randomised controlled trial.^16^ Considering specifically the adaptation of psychosocial interventions for psychosis, there is evidence of increased efficacy of adapted interventions for patients with schizophrenia.^17^

## Cognitive remediation

One such psychosocial intervention for FEP is cognitive remediation, a behavioural training-based intervention that aims to improve cognitive processes with the goal of durability and generalisation.^18^ Cognitive remediation is delivered by a trained therapist and involves the practise of cognitive exercises, with particular attention to the development of cognitive strategies, and procedures to facilitate transfer of cognitive gains to everyday functioning.^18^ It has been demonstrated to provide significant benefits in relation to functioning, cognition and those negative symptoms in schizophrenia,^19^ and has been found to be effective and acceptable for people diagnosed with schizophrenia,^20^ with durable effects.^21^ Computerised delivery of interventions among patients with schizophrenia have been found to be effective and well received,^22^ but still need to be delivered with a trained therapist.^18^ One such example of computerised cognitive remediation is the computerised interactive remediation of cognition training for schizophrenia (CIRCuiTS)^23^ which focuses on metacognition. CIRCuiTS was delivered via internet or CD.^23^ CIRCuiTS is delivered by a therapist, supplemented by independent sessions and is highly acceptable by both patients and clinicians, and feasible for patients with schizophrenia.^24–26^

Given that British South Asian groups have a greater incidence of psychotic illness,^9^ while holding culturally distinct illness beliefs, often following alternative pathways to accessing care, and having specific cultural, religious and linguistic needs,^27^ this group presents an opportunity to examine the benefit of culturally adapting cognitive remediation to improve outcomes for FEP. This is vitally important to address ethnic inequalities in pathways to care. This is demonstrated by the fact that South Asian patients, compared with White patients, have elevated rates of civil detention and repeated admissions.^9^ Specific to early intervention services, there is evidence that British South Asians are more likely to present later to services,^28^ miss out-patient appointments^29^ and are three times more likely to drop out of treatment.^30,31^ Further, British South Asians, like other ethnic minority groups, are often underrepresented in clinical trials for most psychological interventions,^32^ with some tentative evidence that outcomes of standard psychosocial interventions are poorer for ethnic minorities compared with their White peers, as they are underpinned by Western values and norms.^17,33^ Culturally adapted interventions have been shown to be more efficacious compared with usual treatment,^17,34^ and align with National Institute for Health and Care Excellence guidance for schizophrenia, which advocates for ensuring that clinicians and interventions address differing explanatory models, health beliefs and expectations that vary across cultures.^13^

We aim to investigate how to culturally adapt computerised cognitive remediation, and then explore the feasibility of delivering culturally adapted cognitive remediation (caCR) to British South Asians with FEP, but many aspects of this need consideration in terms of feasibility before conducting a randomised controlled trial.^35,36^ Previous research has explored culturally adapting cognitive–behavioural therapy for psychosis,^16^ but this is the first study to assess the feasibility of culturally adapted computerised cognitive remediation. We used two different samples to address this aim, sample 1 (S1) were involved in qualitative interviews to determine how to culturally adapt cognitive remediation, and sample 2 (S2) were involved in exploring the feasibility of the intervention. Assessing feasibility would include exploration of acceptability of the intervention for participants, and if participants are retained in the study.

## Method

### Study design and sample

We employed initial formative qualitative interviews to explore how best to culturally adapt computerised cognitive remediation, followed by a single-group, non-blinded, non-randomised feasibility study with culturally adapted cognitive remediation as the intervention. With respect to the qualitative interviews, we adopted an interpretative approach,^37^ and our sample was generated via purposive sampling of people with FEP from three NHS early intervention services within Lancashire Care NHS Foundation Trust and Manchester Mental Health and Social Care NHS Trust, and non-statutory services in the North-West of England. Early intervention services are constituted by a multidisciplinary team whose objective is to provide support to individuals experiencing a first episode of psychosis. The teams are composed of professionals from a variety of disciplines, including nursing, social work, occupational therapy and psychology. The provision of treatments is consistent across teams where participants were recruited. A total of ten participants were involved in the qualitative interviews. For the feasibility study, our sample was generated via purposive sampling. Researchers contacted community psychiatric nurses (CPNs) and case managers, psychiatrists and clinical psychologists within the Lancashire and Manchester early intervention services, who then made referrals to the research team. A total of ten participants were referred to the research team, and all were eligible for the study and provided written informed consent. Thus, we describe the methods for our two interlinked studies, involving ten participants in each sample (S1 and S2).

### Inclusion criteria

For both the qualitative and feasibility studies, inclusion criteria were age 16–35 years, of self-reported South Asian ethnicity, a DSM-IV diagnosis of non-affective psychosis confirmed with the Structured Clinical Interview for DSM IV (SCID-I/P IV)^38^ and capacity to consent. We focused on 16- to 35-year-olds as computer-based therapy such as CIRCuiTS is valued by younger patients.^25^

### Exclusion criteria

For both studies, exclusion criteria were a DSM-IV diagnosis of substance dependence or misuse, or organic brain disease.

### Procedure

#### Formative qualitative interviews

NHS key workers and voluntary service staff were asked to refer participants that met the inclusion criteria. All participants referred were able to, and provided, informed consent for involvement in the study. Semi-structured interviews were employed, and demographic information was collected at this stage. The interviewer led participants through a 20-min interactive demonstration of the computerised cognitive remediation package CIRCuiTS. This was followed by an interview lasting on average 1.5 h, based around a topic guide, but responsive to issues raised by respondents. The topic guide was developed based on previous literature including author N.H.’s work on culturally adapting psychological interventions, and patient advisors. The guide was informed by the domains commonly explored when assessing acceptability of interventions.^39^ Areas explored by the topic guide included the participants’ concerns with cognition, the acceptability of computerised cognitive remediation and how it could be culturally adapted.

All interviews were recorded and transcribed verbatim. Data was analysed with thematic analysis.^40^ Thematic analysis allows for identifying and describing themes (or patterns of meaning).^40^ Themes were derived inductively; coding and analysis was guided by our study aims on how to adapt cognitive remediation. Coding was an iterative process, developed by moving back and forth through data familiarisation, searching for themes, reviewing themes, defining and naming themes.^40^ These themes were used in conjunction with a framework for cultural adaptation of psychosocial interventions^17^ to implement changes to cognitive remediation.

Participants were encouraged to indicate their preferred language that the intervention be administered, and were able to choose the location for the intervention (this could be at home, or in a public space such as a library). Patients were able to select times that worked around their schedule. One participant exhibited high levels of disorganised and bizarre thought content throughout his interview. The participant’s transcript was independently assessed by two members of the research team (C.P. and R.D.) to identify sections that were too disorganised to be analysed. This process allowed for 63% of the interview to be included in the analysis, rather than removing the entire interview.

### Feasibility study

#### Intervention: computerised cognitive remediation

The intervention was the computerised cognitive remediation programme CIRCuiTS, which is based on paper-and-pencil therapy and was developed with patients and therapists.^25,41,42^ Participants engaged with 40 h of increasingly complex neuropsychological tasks over 12 weeks, and this took place in person. Demographic information was collected. Tasks were delivered by a trained therapist, at the participant’s own pace, typically three to four 1-h sessions per week. CIRCuiTS focuses on developing skills by means of drill, practise and strategy, in common with many other forms of cognitive remediation. The tasks prompt participants to use specific methods (drill); tasks have repeated elements even as they become more complex, so that participants use skills and routines repeatedly (practise); but as tasks become more complex, participants are forced to devise methods by themselves (strategy).^18^ In initial sessions task instructions and the therapist directs participants to use particular strategies, and with increasing task difficulty, the amount of direction decreases, and sessions lengthen as the participants attention and problem-solving skills improve. Correct responses are consistently met with praise from the programme and therapist.

#### Cultural adaptation

We adapted computerised cognitive remediation based on results from our qualitative interviews, and using an established framework.^17^ Our adaptations focused on language, therapeutic alliance, teaching style, manual content, family, treatment goals, delivery and engagement, and stigma. Adaptations are presented in detail in Table 1. Confidentiality was discussed with both the participant and their family.

**Table 1:** Details of the specific components of cognitive remediation and how it was adapted

Aspect of adaptation	Details
Language	All participants were asked about language preference, with sessions being delivered as per patient language preference. For family engagement sessions, all materials were translated into caregivers’ primary language.
Family	A family engagement session invited all family members and the participant to meet with the therapist before the start of caCR sessions. Family members and the participant discussed how cognitive deficits affected their daily activities. A short explanation of caCR was given, and then all members discussed whether they felt the intervention would be beneficial. A key caregiver was identified for weekly contact throughout the intervention. Treatment goals were decided with the family.
Communication	The therapist aimed to provide unconditional positive regard and an encouraging and facilitating atmosphere. The therapist was careful to observe differences in learning style and speed, literacy and computer skills. An example led, practical teaching style was employed. Strategies, developed to complete the tasks more efficiently, were practised every session. Once strategies were confidently used on tasks, the therapist then supported the individual to generalise these strategies to a personalised real-life example within the individual’s cultural context. All participants were encouraged to reflect on their own progress.
Content	All non-halal items were omitted, such as a shopping task that required participants to buy white wine and bacon. Culturally specific foods, geographical knowledge, and social and religious activities replaced or were added to tasks. For example, capital cities had to be learnt in one task, which we changed from European to Islamic countries.
Cultural norms and practises	Supported by evidence from the qualitative study, the therapist was not ethnically, culturally or gender matched. Confidentiality was discussed and ensured. Participants and their families’ illness beliefs and help-seeking pathways to care were openly discussed, but never challenged.
Context and delivery	Frequency and location were completely flexible to the participant’s schedule. Text reminders were sent before every appointment. Those without the internet were provided with paper homework.
Therapeutic alliance	Participants and their families were consulted by their CPNs to confirm this was agreeable. To improve therapeutic rapport, engagement and satisfaction, a flexible schedule always allowed time for informal conversation between the therapist and the family, allowing the therapist to demonstrate cultural competence as appropriate.
Treatment goals	Treatment goals were discussed with the family during the preliminary engagement session. Targets were then reviewed every session between the therapist and participant. On the final session, participants were presented with a certificate; where appropriate, with their families present.

CPN, community psychiatric nurse.

#### Outcome measures

We employed a variety of outcome measures to explore perceived efficacy, acceptability and impact on mental state and neurocognition.

#### Cognitive remediation satisfaction

The Cognitive Remediation Satisfaction Questionnaire^43^ was used to explore participants’ attitudes toward the content of caCR, the computerisation of tasks, rapport with their therapist, self-perceived cognitive effects and any perceived adverse effects. This was considered an assessment of acceptability as domains explored match onto the different component constructs: affective attitude, burden, ethicality, intervention coherence, opportunity costs, perceived effectiveness and self-efficacy.^39^

### Secondary outcomes

#### Mental state

We measured mental state using the following instruments: Positive and Negative Syndrome Scale (PANSS),^44^ Psychotic Symptom Rating Scale (PSYRATS),^45^ Calgary Depression Scale for Schizophrenia (CDSS),^46^ Clinical Global Impressions Scale (CGI)^47^ and Global Assessment of Functioning (GAF), including the Social and Occupational Functioning Assessment Scale (SOFAS).^48^

#### Neurocognition

We measured difference aspects of cognition. Visual learning and memory was assessed via the Ray Osterteith task, immediate recall and long term.^49^ Verbal learning was assessed via the Logical Memory Test.^50^ Speed of processing, an aspect of working memory was assessed via the Trail Making Test (A and B).^51^ Attention and working memory was measured by the N-Back continuous test.^52^ Executive functioning and metacognition were assessed by the Meta-cognitive Wisconsin Card Sorting Test (MC-WCST).^53^ Finally, reasoning and problem-solving was measured by the Wechsler Adult Intelligence Scale Test of Adult Reading (WAIS-R).^54^

#### Other

We also administered the following measures: Beck Cognitive Insight Scale (BCIS),^55^ Birchwood Insight Scale (BIS),^56^ Rosenberg Self-Esteem Scale (RSES),^57^ Brief Illness Perception Questionnaire (IPQ-Brief)^58^ and quality of life via the EuroQol visual analogue scales (EQ-VAS).^59^

### Procedure

All assessment measures, including demographic information, were obtained at baseline apart from satisfaction. Demographic information for S1 included gender, age, ethnicity, country of birth, immigration generation, education level, current employment, household size and whether they were on medication, and was collected before interviews with the use of a self-completed form. For S2, we collected demographic information, including gender, ethnicity, immigration generation, education level, current employment, medication, age and household size. We included more categories of antipsychotics, and age and household size as continuous variables for S2 compared with S1.

There was an initial family engagement session before caCR sessions commenced. Families were invited to meet the therapist. Family members and participants were invited to discuss how cognitive deficits affected their daily activities. A short explanation of caCR was given, and questions answered. A key caregiver was identified for weekly contact throughout the intervention, and this involved a quick check to determine if they felt things were progressing as expected. Participants then undertook 40 h of caCR over 12 weeks. Upon completion, all assessments were repeated, with the exclusion of demographic details and the SCID-I/P IV, and inclusion of the Cognitive Remediation Satisfaction Questionnaire. We primarily determined the feasibility, not efficacy of cognitive remediation. The Cognitive Remediation Satisfaction Questionnaire was completed without contact from the therapist. Questionnaires were left on the last session to be filled in by the participants (with help from family or their CPN where necessary), sealed and then posted back or collected by the research team. All other assessments were carried out by the therapist at baseline and post intervention.

### Analysis

Data was analysed, and continuous pre- and post-intervention data was compared with Wilcoxon signed-rank tests.

### Ethics

The authors assert that all procedures contributing to this work comply with the ethical standards of the relevant national and institutional committees on human experimentation and with the Helsinki Declaration of 1975, as revised in 2008. All procedures involving human patients were approved by the Bolton Research Ethics Committee (qualitative study: reference 09/H1012/2; feasibility study: reference 08/H1009/76).

## Results

### Formative interviews

The participants chose to conduct the interviews in English and in their own homes. Code saturation was achieved after seven interviews, but recruitment continued to total ten participants from the NHS early intervention service. No participants from voluntary services met the SCID-I/P IV criteria. Participant demographics for S1 are presented in Table 2. All participants had experienced their first episode of psychosis within the past 18 months (reflecting the nature of early intervention service programming); eight without relapse and two relapsing once. The mean age was 23.3 years (range 16–30 years); five lived with their parent/s and siblings, three with their spouse and young children, one in council supported accommodation and one in an Islamic residential home. All stated they were Muslim and grew up within an Islamic household.

**Table 2:** Sample characteristics for the qualitative study (S1)

Demographics	*n*
Gender	
Male	6
Female	4
Age, years	
16–19	3
20–23	2
24–27	2
28–30	3
Ethnicity	
British Asian	2
British Pakistani	3
Asian	1
Indian	2
Pakistani	2
Country of birth	
UK	7
Pakistan	2
Bahrain	1
Immigrant generation	
First	4
Second	4
Third	2
Education level	
High school	5
Further education	5
Higher education	0
Current employment	
Unemployed	7
Student	3
Number in household	
3	1
4	2
5	4
6	0
7	1
Supported accommodation	2
Medication	
Atypical antipsychotic	9
No antipsychotic	1

The analysis of the data revealed themes relating to the importance and need for interventions to address cognitive impairment, and then how best to culturally adapt cognitive remediation, in particular, (a) how to adapt the content of the tasks, (b) how best to deliver cognitive remediation and (c) how to make it more engaging. Quotations from interviews relating to the below themes are presented in Table 3.

**Table 3:** Quotations from qualitative interviews

Theme	Quotations
Concerns with cognitive impairment	G: ‘If I’m doing something, like fully concentrating on things, and someone interrupts me, I can’t concentrate any more. Because of this, my mood can change and I don’t like anything. Even can’t concentrate on a film. Two things, I can’t concentrate on at a time.’ D: ‘I miss so many appointments. I don’t remember I’ve got them. If I’ve already made an appointment, I’ll just book them anyway.’ D: ‘I just totally forget that I’m actually cooking. And I’ll leave everything on and start doing something else and then when it’s burnt I’m like, s**t, was I cooking. Then I remember.’ I: ‘I would say a year ago my memory was better. But now it’s a bit weak you know. I do forget things: for example, when I’m playing pool; I forget what my colour is. My mate gets angry. I would say it’s weaker than it used to be; maybe it’s because of the medication.’
Culturally adapting – content	H: ‘Muslims don’t eat *Haram* food, it’s meat mostly, and gelatine.’ E: ‘You can include local dish…garam masala, fresh chilli.’ G: ‘It’s quite different for me, because I’m not familiar with this kind of recipes and these things [ingredients].’ I: ‘I’d change the church to a mosque. And the dressing, I’d change it to more what we would wear, more Asian type.’
Culturally adapting – engagement	B: ‘I was really conscience of what everybody would think about me and like if everybody found out I was in hospital, what they would be thinking about me.’ D: ‘My husband, he’s brought someone home as well…He believes in that guy really strongly. What they do basically, you’ve got like a spirit inside you…He can basically talk to *jinns*.’ H: ‘What I’m going through affects my family as well, so I think I would want them to get some help as well.’

#### Perspectives of cognitive remediation

All respondents expressed their frustration with the effect of cognitive impairments on their social functioning, meaningful daily activities and self-confidence. All wanted to improve their cognitive skills. Computerised cognitive remediation was well received by all participants: ‘interactive’, ‘colourful’ and ‘fun’. Respondents were able to offer advice about the delivery and content of cognitive remediation.

#### Content of cognitive remediation tasks

Participants suggested making the content of the tasks more culturally familiar (i.e. include capital cities from Muslim countries, Asian names, ingredients for recipes) and religiously sensitive (i.e. remove all non-*Halal* foods from shopping or cooking tasks).

#### Delivery

Most respondents preferred cognitive remediation sessions to take place in their own home, saving them time and avoiding costs for travel and childcare. Those unsure were concerned they would be distracted by younger family members during sessions. Field notes highlight the presence of family members during all interviews, often young children needing attention from the participant, or older family members who spoke little English but expressed an interest in the nature of the intervention. All participants suggested a high frequency of sessions was preferable, between twice and five times a week.

The majority of participants had their own home computer and regularly accessed the internet, but some had limited or no access and wished to improve their computer skills. All participants preferred computerised cognitive remediation to the idea of using pen and paper, but many asked for clearer instructions to the programme and a preliminary session to familiarise themselves with the computer.

All considered the support of a therapist essential during sessions. All but one preferred working one to one with the therapist rather than in a group. This individual was attracted by the social aspects of a group. Emphasis was placed on the competence and amiability of the therapist, whereas ethnicity or specific qualifications were dismissed as unimportant. Two individuals (one female, one male) preferred the therapist to be gender matched.

All participants preferred the cognitive remediation programme to be in English, but recommended that a translated version should be available. Only one bilingual participant encountered linguistic problems on one task. This required participants to respond quickly to an image on the screen by pressing the letter it began with on the keyboard. He explained that the additional cognitive process of translating what he had seen appeared to increase his reaction times.

#### Engagement

Participants highlighted the importance of being receptive to participants’ holding multiple or distinct illness beliefs, openness to help-seeking from multiple sources, and awareness of problems concerning confidentiality and community stigma. Respondents highlighted the importance of family: not only providing families with clear information, but recognising their willingness to help their family member. There was also a clear requirement for therapists to exhibit cultural competence and recognise the individuals’ and their families’ level of acculturation and language needs.

### Feasibility study

#### Sample and retention

Sample characteristics for S2 are presented in Table 4 for our feasibility study. The mean age was 24, the sample was evenly divided by gender and most identified as Pakistani. A majority of the sample were second generation immigrants, and only one participant was not on antipsychotic medication. Two participants withdrew; both were full-time students. One withdrew immediately after baseline assessments: they felt they did not suffer from cognitive deficits, so caCR would not be of any benefit. The other completed four sessions of caCR, but being in their final year of university, said they did not have time to continue with sessions.

**Table 4:** Sample characteristics for feasibility study (S2)

Characteristics	*n* (%)
Categorical variables	
Gender	
Male	5 (50%)
Female	5 (50%)
Ethnicity	
British	1 (10%)
British Asian	2 (20%)
British Pakistani	1 (10%)
Indian	1 (10%)
Pakistani	5 (50%)
Immigrant generation	
Second	9 (90%)
Third	1 (10%)
Education level	
High school completed	10 (100%)
Further education	10 (100%)
Higher education	0 (0%)
Current employment	
Unemployed	7
Student	3
Medication	
Aripiprazole	3 (30%)
Clozapine	1 (10%)
Olanzapine	3 (30%)
Quetiapine	1 (10%)
Risperidone	1 (10%)
No medication	1 (10%)
Continuous variables	Mean (s.d.)
Age, years	24.7 (4)
Number in household	5.4 (0.84)

#### Acceptability (the Cognitive Remediation Satisfaction Questionnaire)

##### Sessions

Overall, our participants reported that the tasks were fun and challenging. Seven (87.5%) enjoyed the attention they got during therapy, six (75%) said sessions kept their mind occupied, and seven that there were the right number of sessions per week. With one exception, all participants completed 40 h of caCR. The participant who did not complete the caCR had fluctuating, severe symptoms that made engagement intermittent and therefore prolonged the intervention. For this individual, only 20 h of caCR was completed over 6 months. Scheduling of sessions was flexible, as several participants attended college full time or had young children. The majority preferred two 1.5–2 h sessions per week. All sessions took place at the participant’s family home, with the exception of one participant, who asked for sessions to move to the local library during school holidays, to allow them to concentrate away from younger siblings. To improve participant and family engagement, the therapist tried to avoid seeming hurried and always left time at the beginning and end of sessions for informal conversation with families and participants. The majority (87.5%) were disappointed when sessions ended. Reasons given included that they would miss their therapist, found caCR beneficial and worthwhile, and enjoyed the challenge and the progressively increasing difficulty. One respondent indicated relief when sessions ended because caCR had gone on too long, but added that they would miss contact with the therapist. Further, 50% of our sample said they had improved their computer skills during sessions.

##### Tasks

Our sample was divided into those who did (*n* = 4) and did not (*n* = 3) find the tasks difficult, and there was no consensus on which task was the most difficult. The majority who found it difficult conveyed they enjoyed the challenge. Among our sample, seven of the eight participants felt they had improved as the therapy proceeded, and all reported this had made them feel better. For those who did not improve, one participant found it frustrating. Each participant named a different task as their favourite, and all but one said they understood the use of strategies for each task. Four participants stated they had used the tasks and strategies outside the therapy sessions. This included using lists and memory strategies in the supermarket, working out travel times for transport, planning and to-do lists, and reasoning strategies to make decisions. Three participants had never used strategies outside of caCR sessions.

##### Family

For seven of the families, the therapist engaged with at least one family member throughout the intervention. All participants stated family members understood why the therapist visited, and all but one said that their family was happy they were participating. The exception, whose family had limited engagement during the intervention, said he was uncertain. For three families, despite many efforts, the therapist was only able to make contact with the primary caregiver on the preliminary engagement session.

##### Therapeutic alliance

All participants assessed the therapist positively and identified qualities such as being a good teacher, being non-judgemental, easy to talk to and encouraging. Several participants reported the most helpful attribute of cognitive remediation sessions was having someone to talk to. The therapist and the participants were not ethnically matched. Religion (specifically Islam), spirituality and British South Asian cultural norms were frequently discussed. Families often sought some disclosure of the therapist’s own cultural background. The therapist encouraged the family to advise them on cultural norms, and spent a considerable amount of time exploring the families’ treatment expectations and goals.

##### General benefits and costs of caCR

Although four (50%) reported caCR had made them more aware of their limitations, none found this frustrating. Seven (87.5 %) said caCR made them feel better about themselves, boosted their confidence, improved their mood and increased their general knowledge; that they enjoyed using strategies to complete the harder levels and that it gave them an understanding of metacognition. Helpful outcomes included improvements in memory, having someone to talk to, occupying spare time, improving mood and the real-life application of strategies. The only negative outcome was the frustration when getting tasks wrong and exposing their cognitive limitations.

##### Specific aspects of cognition

Self-perceived changes in concentration, memory and alertness are displayed in Table 5. Among our sample only one out of the eight (12.5%) participants felt that concentration, memory and alertness remained a problem after the intervention. Two out of eight (25%) participants reported permanent improvement in concentration, three out of eight reported permanent improvement in memory and three out of eight reported permanent improvement in alertness, and the rest reported that the intervention helped at the time.

**Table 5:** Self-perceived changes in concentration, memory and alertness

Aspect of cognition	Perception, *n* (%)		
	Still a problem	Helped at the time	Helped permanently
Concentration	1 (12.5%)	5 (62.5%)	2 (25%)
Memory	1 (12.5%)	4 (50%)	3 (37.5%)
Alertness	1 (12.5%)	4 (50%)	3 (37.5%)

#### Secondary measures

##### Mental state

Even with this small sample, there were significant improvements (uncorrected for multiple testing) in PANSS and PYSRATS totals and GAF and SOFAS scores (Table 6). No significant difference in baseline compared with post caCR were identified for health-related quality of life (EQ-VAS), beliefs about illness (IPQ-Brief), cognitive insight (BCIS), insight (BIS), depression (CDSS) and self-esteem (RSES).

**Table 6:** Secondary outcomes before and after culturally adapted cognitive remediation

Secondary outcomes	Baseline mean (s.d.)	Post-caCR mean (s.d.)
Mental state		
PANSS Positive	14.0 (4.8)	12.5 (4.0)
PANSS Negative	15.4 (6.28)	14.3 (2.7)
PANSS General Psychopathology	32.2 (13.4)	28.9 (7.5)
Total PANSS**	61.3 (20.1)	55.6 (11.5)
Total PSYRATS	18.7 (17.9)	17.0 (14.0)
CDSS	10.9 (6.1)	8.9 (6.6)
CGI	3.2 (0.4)	3.6 (1.1)
GAF**	53.0 (13.2)	58.4 (10.7)
SOFAS*	54.2 (13.8)	58.0 (10.1)
Other		
BCIS	10.1 (6.8)	7.8 (7.7)
BIS	10.1 (2.9)	10.8 (1.6)
RSES	24.9 (3.7)	25.1 (3.3)
IPQ-Brief	152.9 (14.7)	159 (21.9)
EQ-VAS	64.5 (18.2)	68.8 (18.5)
Neurocognition		
Visual learning and memory		
Ray Osterieth task immediate recall*	32.0 (3.3)	33.3 (2.3)
Ray Osterieth long term**	14.2 (5.8)	24.9 (6.2)
Verbal learning and memory		
Logical memory immediate recall**	7.1 (3.5)	11.2 (6.1)
Logical memory recall**	6.2 (3.8)	9.1 (5.2)
Speed of processing		
Trail Making Test A	45.5 (24.3)	29.9 (31.9)
Trail Making Test B*	89.4 (52.3)	72.2 (51.8)
Attention		
N-back total (0 back)	5.9 (0.3)	5.1 (0.3)
Working memory		
N-back (1,2,3 back)	12.6 (12.3)	12.0 (3.2)
Metacognition		
MC-WCST	0.1 (0.2)	0.1 (0.1)
Reasoning and problem-solving		
WAIS-R**	31.6 (14.1)	39.1 (14.9)

caCR, culturally adapted cognitive remediation; PANSS, Positive and Negative Syndrome Scale; PSYRATS, Psychotic Symptom Rating Scale; CDSS, Calgary Depression Scale for Schizophrenia; CGI, Clinical Global Impressions Scale; GAF, Global Assessment of Functioning; SOFAS, Social and Occupational Functioning Assessment Scale; BCIS, Beck Cognitive Insight Scale; BIS, Birchwood Insight Scale; RSES, Rosenberg Self-Esteem Scale; IPQ-Brief, Brief Illness Perception Questionnaire; EQ-VAS, EuroQol visual analogue scales; MC-WCST, Meta-cognitive Wisconsin Card Sorting Test; WAIS-R, Wechsler Adult Intelligence Scale Test of Adult Reading.

*P < 0.05; **P < 0.01.

##### Neurocognition

Global cognitive change had an effect size (Cohen’s *d*) of 0.40. Learning and memory showed evidence of appreciable improvement (effect sizes of 0.46 and 1.77) for visual immediate and long-term recall (Rey Osterieth task), verbal immediate and long-term recall (Logical Memory Task, effect sizes of 0.83 and 0.63, respectively), and reasoning (WAIS-R) (effect size 0.52). Improvements in speed of processing (Trail Making Test A, 0.20; Trail Making Test B, 0.33) was close to expected practice effects. Working memory and metacognition’s effect sizes were 0.

## Discussion

### Cultural adaptation

Computerised cognitive remediation was well received by all respondents, who recommended modifications to the content and mode of delivery. We used these findings from our thematic analysis in conjunction with another model for culturally adapting interventions.^17^ Our adaptations focused on language, family, communication, content, cultural norms and practises, context and delivery, therapeutic alliance and treatment goals. As mentioned, all of the sample stated they were Muslim, and this therefore influenced the adaptions made to the cognitive remediation, such as incorporating *Halal* foods. We did not focus on adapting cognitive remediation with respect to concepts and illness models, as this did not arise as an issue during our qualitative interviews. Participants’ recommendations largely fit within Bernal and Sáez-Santiago’s existing theoretical framework for culturally adapting psychological interventions for ethnic minorities.^60^ The model recommends modifying language, culturally matching client, and therapist, using culturally familiar content and establishing common treatment goals between the patient and provider. The framework was based on research exploring the needs of Latin Americans. Notable differences between the model and this study’s results were clients’ preference for conversing in the English language and an apparent indifference to the therapist’s ethnicity. These differences are important for practise, implementation and scale-up.

Other results are consistent with adaptations made in previously published randomised controlled trials of adapted interventions for psychosis. These include incorporating cultural and religious content;^61,62^ acknowledging distinct illness beliefs;^63,64^ recognising the importance of family, specifically a distinct family structure or hierarchy;^65–67^ recognising patient and family expectations and treatment goals;^65,67^ the importance of confidentiality, given patients’ perception of stigmatisation by their community and families;^65^ and optimising delivery, i.e. flexibility to financial, domestic or religious needs.^67,68^

### Feasibility outcomes

Results from the satisfaction questionnaire showed that caCR was highly acceptable as an intervention to this sample of British South Asian with FEP. Levels of engagement and satisfaction were high. Enthusiasm and engagement were determined by family engagement, the challenge of the tasks and therapist contact. We identified that the family was central to optimising delivery and engagement. Other studies have identified the importance of family engagement for success of psychosocial interventions.^69^ Participants for which contact with families was limited were most likely to drop out, cancel sessions or report they had improved the least. Family members, particularly young children or siblings, were frequently at home throughout caCR appointments and would often want to sit in on sessions. During the preliminary family engagement session, family members frequently identified cognitive deficits and discussed how this affected the whole family. They were keen to have an active role: setting aside a quiet room during sessions, motivating participants to complete homework and discussing the goals of the intervention. Previous studies focusing on British South Asians have stressed the importance of a good relationship between the therapist/facilitator and the patient’s family.^70,71^ Future studies could explore this further to determine the critical ingredients in relation to family involvement.

It has been evidenced that issues with transport and childcare can be a deterrent for engaging with psychosocial interventions,^70,72^ therefore it is likely that the flexibility of the therapist to adjust to participants’ schedules, and being able to visit them at home, kept participants engaged. Participants enjoyed having someone to talk to, and to keep them motivated throughout sessions. All of the participants were young; many were still or had just recently stopped studying. All commented positively on how CIRCuiTS sessions reminded them of studying.

With respect to retention, two of the initial ten participants dropped out. However, baseline cognitive data and verbal feedback suggests neither participant dropped out because of severe cognitive impairment or dissatisfaction with the intervention; however, both were busy students, and this may reflect that this intervention is more of a challenge to engage with if the participant has a busy schedule. Given that patients with psychosis are often hard to engage, the attrition rate of this intervention is low, with 80% of our sample completing 12 weeks of cognitive remediation and high levels of engagement.

### Strengths and limitations

One strength of this study is that we ensured purposive sampling was from three areas that support large ethnic minority communities, similar to many urban areas in the UK. Barriers to recruitment were minimised by asking language preference, choice of location and time of interview, exhibiting cultural competence and reimbursement of all costs. Our intervention was delivered in English and we recognise that not translating the intervention is a limitation. A future trial would be designed to consider preferred language and translate the intervention, similar to the approach used in ROSHNI-D study.^73^ The sample varied in gender, age, source of income, previous convictions, level of education, immigrant generation, family structure, acculturation and religious beliefs. This increased our confidence that the saturation of key themes was valid in the qualitative interviews.

However, although our study offers a novel insight into how to culturally adapt cognitive remediation and the feasibility of such an intervention, there are important limitations to this study. Our study has a small sample size and as such results should be interpreted with caution. Our sample was entirely Muslim, mostly Pakistani and born in the UK, and therefore not reflective of all British South Asians. The analyses of our feasibility study were exploratory, with significance uncorrected for multiple testing. Without a control group, assessments were not blind, and we are unable to determine if improvements in mental state and cognition are better than usual cognitive remediation/routine care. Further feasibility data, including deriving eligible numbers to power a randomised controlled trial and assessment of willingness to be randomised, is needed to fully satisfy that this intervention can be employed in a trial and allow for the development of a protocol. Future feasibility work should also focus on determining if caCR is more or less effective depending on psychosis symptoms and severity.

In conclusion, this study is the first study that provides novel evidence on how to culturally adapt cognitive remediation for Muslim British South Asians with FEP in the UK. We have demonstrated this caCR is highly acceptable, with high levels of satisfaction and engagement and good retention. This study indicates that computerised cognitive remediation can be successfully culturally adapted for British South Asians with FEP, and such an intervention is acceptable. Further feasibility evaluation is needed, such as assessing agreeability for randomisation and estimation of effect sizes for future randomised controlled trials.

## Data Availability

The data that support the findings of this study are available from the corresponding author, J.B., upon reasonable request.
